# The Effect Of Localized Circumferential Residual Stress On Pressurized Stress State Of CT Reconstructed Vessels: A Finite Element Study

**DOI:** 10.1007/s10439-026-04085-x

**Published:** 2026-03-23

**Authors:** Vittorio Lissoni, Martina Schembri, Giulia Luraghi, Alessandro Caimi, Virginia Fregona, Mauro Di Giancamillo, Jessica Bassi, Ferdinando Auricchio, Michele Conti, Gabriele Dubini, Francesco Migliavacca, Jose Felix Rodriguez Matas

**Affiliations:** 1 LabS-Laboratory of In Silico Medicine, Department of Chemistry, Materials and Chemical Engineering “Giulio Natta”, Politecnico di Milano, Piazza Leonardo da Vinci 32, 20133 Milano, Italy; 2 Civil Engineering and Architecture Department, Università Pavia, Pavia, Italy; 3 Department of Veterinary Medicine and Animal Sciences, Università degli Studi di Milano, Milan, Italy; 4 Foundation IRCCS Ca’ Granda Ospedale Maggiore Policlinico, Milan, Italy; 5 3D and Computer Simulation Laboratory, IRCCS Policlinico San Donato, San Donato Milanese, Italy

**Keywords:** Residual stress, Aorta, Finite element model, Segmentation, Mechanical properties

## Abstract

**Purpose:**

Residual stresses are intrinsic stresses present in arteries even in the absence of external loads or blood pressure. They originate during growth and remodeling and contribute to vascular mechanical equilibrium. Despite advances in computational methods, incorporating residual stresses into patient-specific vascular models remains challenging. Evidence regarding the influence of circumferential residual stresses on physiological loading in realistic anatomies is still limited.

**Methods:**

We combined *in vitro* imaging, mechanical testing, and finite element (FE) simulations to incorporate residual stresses into a pressurized porcine aorta model. Medical images were acquired in both pressurized and zero-stress states, the latter obtained after 2 h of longitudinal cutting. Locally measured mechanical properties from distinct aortic regions were used to define an orthotropic hyperelastic material response. Residual stresses were restored by simulating vessel closure under displacement-controlled boundary conditions with a tied contact interface. Stress distributions were then compared in FE pressurization simulations performed with and without residual stresses.

**Results:**

Residual stresses substantially influenced the predicted stress fields. They homogenized stress across the vessel thickness and reduced stress concentrations near the lumen. Moreover, lumen cross-sectional areas at 120 mmHg aligned more closely with experimental measurements when residual stresses were included. In contrast, neglecting residual stresses produced an average overestimation of lumen area by 4.84% and stress peaks up to 50% higher in the inner wall.

**Conclusion:**

This study highlights the importance of accounting for residual stresses in biomechanical vascular simulations. Incorporating them improves prediction accuracy and reduces artificial stress concentrations, which is crucial for patient-specific risk assessment and surgical planning.

## Introduction

Residual stresses are internal stress states that persist within blood vessel walls. They arise during the natural processes of vascular growth and remodeling, primarily due to the non-uniform deposition of structural components such as elastin and collagen [1, 2]. Since these stresses are inherently linked to the vessel’s microstructural organization, they remain present even when the vessel is in an unloaded and unpressurized state.

Their existence was first demonstrated by Chuong and Fung through the ring-opening test, where a radially incised arterial ring opens into a sector, revealing the presence of residual circumferential stress [3]. Since these early studies, numerous *in vitro* experiments have been conducted to further characterize residual stresses in both human and animal arteries. These investigations have shown that residual stresses manifest not only circumferentially, as seen through the opening of arterial rings, but also longitudinally, as excised vessel segments tend to shorten upon release from surrounding tissue constraints, due to the so-called axial pre-stretch as first reported by Vassoughi [4]. Regarding the stress analysis in vascular tissue, Vaishnav *et al.* [5] are among the first to develop a method for determining the distribution of circumferential, longitudinal, and radial stress accounting for through the thickness of the aortic wall under physiological loading in dogs. They assumed the aortic tissue as homogeneous, incompressible, elastic, and circumferentially orthotropic. However, they did not considered the residual strains in the tissue which were not considered. A subsequent work by Holzapfel *et al.* [6, 7] made significant advances in capturing layer-specific stress distribution in coronary arteries accounting for residual stresses. In their 2007 study [6], longitudinal and circumferential samples of the abdominal aorta were excised from 11 human cadavers, and the stress-free configurations of the individual arterial layers (intima, media, adventitia) were determined. These data were later used to formulate a three-dimensional analytical model for predicting residual stress distribution in heterogeneous arterial tubes [7]. Their results featured the compressive nature of the circumferential stress in the intima, and the linear variation of the circumferential stress in the media (compressive at its inner boundary and tensile at its outer boundary). Another important contribution to the experimental characterization of both circumferential and axial residual stresses was made by Sokolis *et al.* [8–11] and axial pre-stretches were investigated by Horny *et al.* [12–14]. Through detailed mechanical testing of human aortic segments, Sokolis *et al.* demonstrated that residual stresses are highly location specific along the aorta. In particular, circumferential residual strains vary significantly from the proximal to distal segments of the descending aorta [7], while axial residual strains were shown to increase in the abdominal aorta and to exhibit layer-specific and quadrant-dependent variations around the vessel circumference [11]. Horny *et al.* [13, 14] and Sokolis *et al.* [11] showed, respectively, the axial pre-stretches and axial residual strains to be age-related. Sex was also demonstrated to be an aspect influencing residual stresses [10]. All this evidence concurs in affirming the patient specificity of residual stress magnitudes.

Beyond merely confirming their existence, understanding the role of residual stresses is critical for the accurate interpretation of computational biomechanics studies, especially in patient-specific simulations. In this regard, Wang and Gleason [15], in a study conducted on the left anterior descending coronary artery and the common carotid artery of the pig, further demonstrated the importance of residual strains in particular the role of longitudinal residual strains in the stress analysis of arteries. They showed how the inclusion of the longitudinal residual strain further uniformizes the circumferential stress gradient through wall while increasing the axial stress gradient through wall up to sevenfold. In silico models usually rely on anatomical reconstructions derived from *in vivo* imaging, in which the vessel is affected by residual stresses other than the pre-stress state caused by the internal pressure. While axial pre-stretches can be introduced by axially pre-stretching the segmented geometry [16, 17], determining the real stress-free configuration, particularly with respect to circumferential and axial residual stresses, is not directly feasible for non-cylindrical geometries from imaging data [17]. Consequently, most computational studies [7, 18–23] have relied on analytical models to estimate circumferential residual stresses rather than attempting to model the opening process or extract stress information directly from imaging. The approach proposed by Holzapfel *et al.* [7] remains the mostly used, combining *in vitro* measurements of the opening angles with mechanical modeling to reconstruct the residual stress field. Studies such as those by Pierce *et al.* [18], Alastruè *et al.* [24], and Raghavan *et al.* [25] have applied this approach to human patient-specific models, while others, including Zhang *et al.* [21], Sigaeva *et al.* [20], and Breslavsky *et al.* [22], have focused on idealized cylindrical geometries. Few studies as in Raghavan *et al.* [25] and in Labrosse *et al.* [26], among others, estimate residual stresses by simulating the closure of the open vessel through displacement boundary condition on idealized cylindrical vessels.

Nevertheless, the strong location and patient specificity of residual stresses, as emphasized by Sokolis [9, 11] and Horny [14], suggests that generalized analytical models may fail in capturing the full complexity of arterial biomechanics, especially in anatomically realistic or pathologic scenarios. Many prior studies relied on simplified cylindrical geometries, potentially overlooking critical anatomic variations that influence stress distribution [24, 25]. The use of generalized analytical models and the use of oversimplified geometries represent important limitations in the investigation of residual stresses proposed in the literature. On top of that, in most of the studies, residual stresses were modeled but the effective difference in the stress state accounting and not accounting for the residual stress was quantified only in Labrosse *et al.* [26] on an idealized cylindrical sample.

In this study, we aim to overcome these limitations by quantifying the circumferential residual stresses and evaluating their impact on vessels stress predictions under physiological conditions using finite element (FE) simulations. To this end, we conducted an *in vitro* experiment using a porcine aorta. The vessel was cut longitudinally to release circumferential residual stresses, and the open configuration was captured using computed tomography (CT). Longitudinal and circumferential tissue strips were excised and mechanically tested to determine local material properties. A segmentation-based model of the open aorta was then used in FE simulations to recreate the closure process and reintroduce residual stress. Subsequently, physiological pressure was applied to the closed configuration both accounting and not for the obtained residual stresses. The resulting stress distributions were analyzed to quantify the influence of residual stress inclusion on the biomechanical response of the vessel wall. Axial residual strains were not incorporated in the present framework due to the impossibility of having a reference configuration prior the excision of the aorta.

## Materials and Methods

### Aorta Preparation

The studied aorta (from the aortic annulus to the celiac trunk) was harvested from a *hybrid large white pig* in a local slaughterhouse. Since the vessel was collected from animals that have already been butchered, only generic values of weight (160–180 kg) and age (11–12 months) can be reported. The animals were raised and sacrificed for commercial purposes and not for this study. The sample was surgically prepared by removing extra connective and cardiac tissues and ligating the supra-aortic branches and spinal arteries and then tested within 5 h from the harvesting to ensure none or minimal tissue decomposition. For the image acquisition, the aorta was secured at the container at both proximal (*i.e.,* aortic annulus) and distal (*i.e.,* celiac trunk) extremities and suspended in water.

**Fig. 1:**
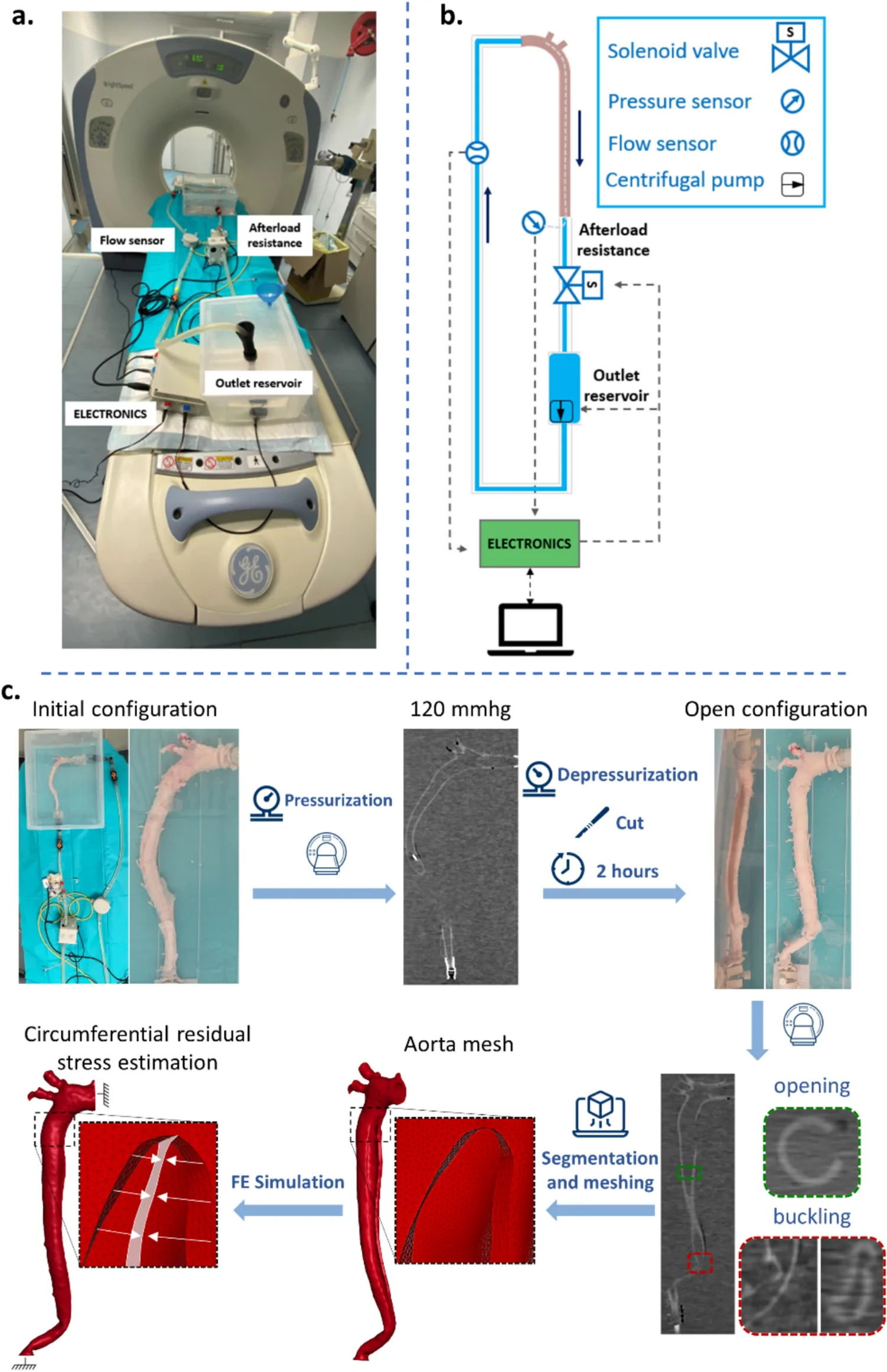
**a** Photo of the portable Mock Circulatory Loop suitable to perform CT acquisition of the aorta sample. **b** Scheme of the MCL. An Arduino UNO microcontroller was used to acquire pressure and flow signal and to control the RPM of a centrifugal pump to generate continuous flow and the proportional closure of a solenoid valve to regulate the pressure values. **c** Experiment carried out on the aorta starting from the pressurization up to 120 mmHg and medical image collection of the pressurized shape, cut and medical image collection of the open aorta 2 h after the cut, segmentation and meshing of the open aorta, and circumferential residual stresses estimation through numerical simulation of cut closure

The aorta was pressurized up to 120 mmHg through an ad-hoc Mock Circulatory Loop (Fig. 1a), and a medical image of the pressurized configuration was acquired (Fig. 1b)

In particular, as schematized in Fig. 1b, a commercial centrifugal pump (DC 12 V, Qmax 800 L/H) was used to generate a continuous flow of 5 L/min and the pressure values (80–120 mmHg) were regulated thanks to the use of a two-way proportional solenoid valve (Bürkert 2875, Burkert Italia Spa, Milano, Italy), placed downstream the vessels. An Arduino UNO R3 microcontroller (Arduino.cc, Somerville, MA, USA) was employed to acquire pressure and flow signals (pressure sensors: HONEYWELL 40PC Series, Honeywell, Charlotte, USA; flow sensor module: Renesas FS1025-DL, Renesas Electronics Corporation, Tokyo, Japan) and to automatically regulate the revolution per minute (RPM) of the centrifugal pump and the closure of the proportional valve to achieve the correct pressure and flow values.

CT acquisitions were performed using a GE BrightSpeed 16-slice multidetector computed tomography (MDCT) scanner (GE Healthcare, Waukesha, WI, USA), equipped with HiLight Matrix II detectors and Volara digital acquisition system (DAS). The scanner provides sub-millimeter in-plane spatial resolution (up to 0.350 mm), with images acquired in helical mode using a slice thickness of 1.25 mm and a reconstruction interval of 0.625 mm. Acquisition parameters were set to 120 kV, 180 mA, and a pitch of 0.9373. No contrast medium was administered for image collection. Pressure was brought back to 0 mmHg and the aorta was cut longitudinally in the inner quadrant of the aorta using a blade. The cut extended axially from approximately 1 cm distal to the second supra-aortic branch to approximately 2 cm proximal to the celiac trunk. The specimen was then let rest in water for 2 h to release the circumferential residual stress before capturing the medical image of the open configuration. The open configuration of the aorta is shown in Fig. 1c. After 2 h, it is possible to observe not only the opening of the cut but also the bending and lumen closure in correspondence of the distal end of the cut. The image also shows details extracted from the medical image of the opening of the vessel in the midpoint of the cut, of the bending and of the lumen closure.

### Mechanical Properties Characterization

**Fig. 2:**
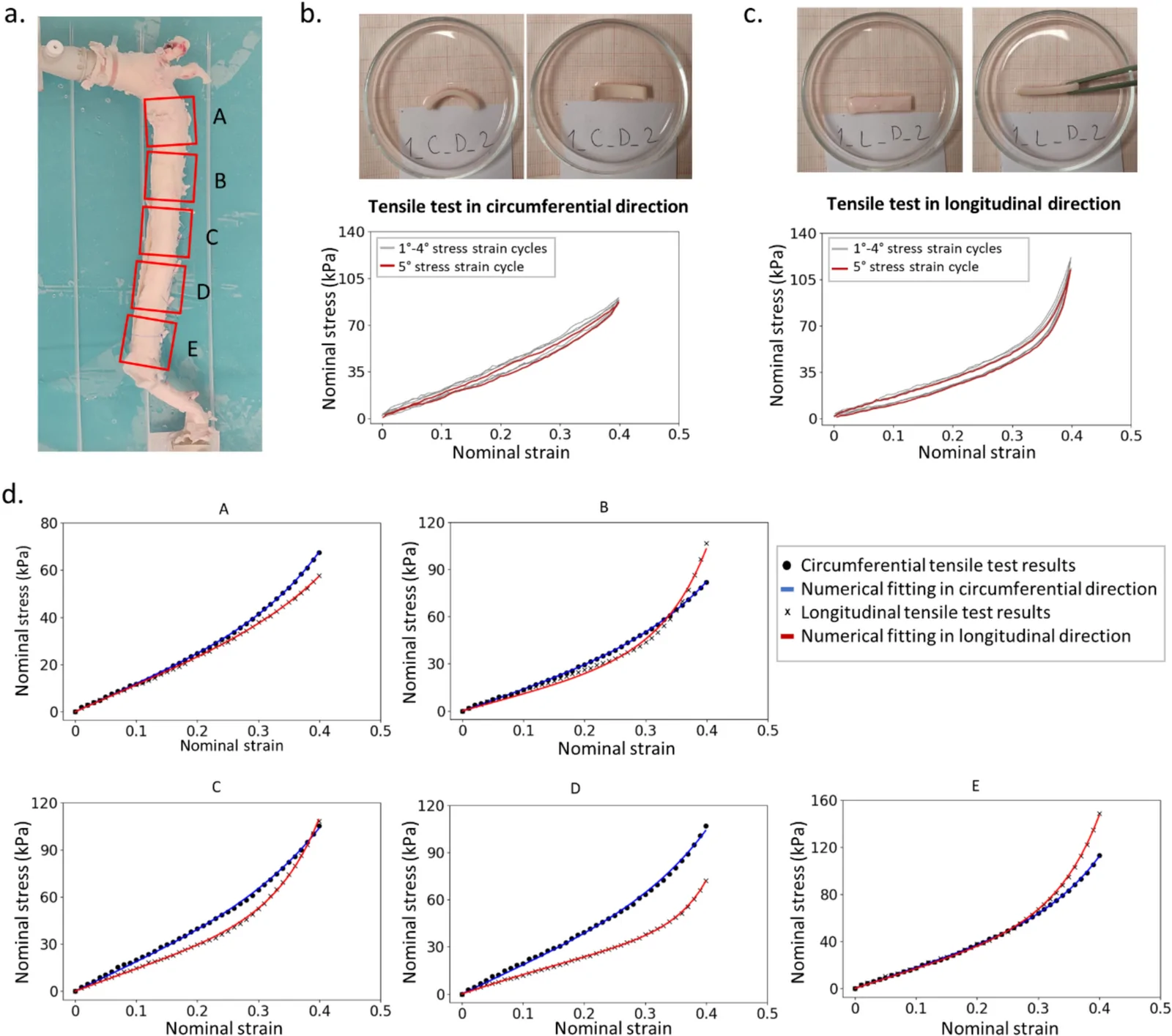
Mechanical characterization of the pig aorta. **a** The five zones where samples were extracted. **b** Example of sample in circumferential direction from section D with the resulting experimental stress–strain curves. **c** Example of sample in longitudinal direction from section D with the resulting experimental stress–strain curve. **d** Numerical fitting of the load curves

From the cut-open aorta, a total of 30 strips were collected: we identified 5 longitudinally progressive zones starting from the proximal descending thoracic aorta and having approximately the same length of 45 mm (identified with the letters A, B, C, D, and E as shown in Fig. 2a). From each anatomical zone, three longitudinal and three circumferential strips were collected using a cutting template (5.0 mm × 25.0 mm). Longitudinal specimens were extracted from the posterior, anterior, and outer quadrants of the aortic wall, whereas circumferential specimens were extracted such that their center was approximately located in the posterior, anterior, and outer quadrants. Specimen thickness was defined as the average of three measurements taken along each sample to account for the non-uniform thickness of the aortic wall. Examples of samples extracted from zone D are shown in Fig. 2b and c, respectively, for circumferential and longitudinal direction. Samples were treated as homogeneous without distinguishing the intima, media, and adventitia layers.

Samples were tested under displacement-controlled uniaxial tension, performing five loading cycles up to 40% strain at a constant displacement rate of 0.10 mm/s. Tests were performed in air, and the specimens were not immersed in any physiological solution or temperature-controlled bath. The specimens were clamped in the testing device, resulting in an effective gauge length of 10 mm and a gauge width of 5 mm, with the thickness measured for each sample. Tension test were performed with a TC-3F Bioreactor from EBERS Medical Technology SL (Zaragoza, Spain) using a 1-lb load cell (Miniature S-beam Jr Load Cell, FUTEK) and processed with EBERS TC software. The resulting force measurements were converted into nominal stress by dividing by the cross-sectional area, which was determined from images of each specimen using ImageJ (National Institutes of Health, USA). The five loading and unloading stress-strain cycles for two samples are shown as example in Fig. 2b and c for the circumferential and longitudinal direction, respectively. For each anatomical zone, the final loading cycle of the three longitudinal and three circumferential specimens was selected. The corresponding stress–strain curves were uniformly resampled and averaged for each loading direction to obtain a representative mean response. The constitutive parameters were identified by performing two independent fitting procedures, one for the longitudinal and one for the circumferential response, while adopting the same strain energy formulation and fitting strategy. The averaged stress–strain curves in the loading region were then used to fit the Holzapfel–Gasser–Ogden constitutive model [27] (Fig. 2c), and to identify the corresponding material parameters, described by the following strain energy function:

1 $${\text{W = C}}_{{{10}}} \left( {{\overline{\mathrm{I}}}_{{1}} { - 3}} \right){ + }\frac{{{\mathrm{k}}_{{1}} }}{{{\mathrm{k}}_{{2}} }}\left( {{\mathrm{e}}^{{{\mathrm{k}}_{{2}} \left( {{\overline{\mathrm{I}}}_{{4}} { - 1}} \right)^{{2}} }} { - 1}} \right){ + }\frac{{{\mathrm{k}}_{{3}} }}{{{\mathrm{k}}_{{4}} }}\left( {{\mathrm{e}}^{{{\mathrm{k}}_{{4}} \left( {{\overline{\mathrm{I}}}_{{6}} { - 1}} \right)^{{2}} }} { - 1}} \right){ + }\frac{{{\mathrm{K}}_{{0}} }}{{2}}\left( {\frac{{{\mathrm{J}}^{{2}} { - 1}}}{{2}}{\text{ - lnJ}}} \right)$$

where ${C}_{10}$, ${k}_{1}$, and ${k}_{3}$, are material constants with units of stress, ${k}_{2}$ and ${k}_{4}$ are dimensionless, and ${K}_{0}$ is the bulk modulus. ${\overline{I} }_{1}$ is the first invariant of the isochoric right Cauchy–Green deformation tensor, $\overline{\mathrm{C} }$, where as ${\overline{I} }_{4}={\mathrm{n}}_{\theta }\bullet \overline{\mathrm{C}}\bullet {\mathrm{n} }_{\theta }$ and ${\overline{I} }_{6}={\mathrm{n}}_{l}\bullet \overline{\mathrm{C}}\bullet {\mathrm{n} }_{l}$ are the pseudo-invariants associated with the circumferential, ${\mathrm{n}}_{\theta }$, and longitudinal, ${\mathrm{n}}_{l}$, directions of the artery respectively, and J is the elastic volume ratio. *K*_0_ values were calculated to guarantee the nearly-incompressibility hypothesis (*ν* = 0.49) with the following formula:

2 $$K_{0} = \frac{{4 C_{10} \left( {1 + \nu } \right)}}{{3 \left( {1 - 2\nu } \right)}}$$

In the longitudinal fitting procedure, only the parameters *k*_1_​ and *k*_2_​ were identified, whereas in the circumferential fitting procedure only *k*_3_​ and *k*_4_​ were fitted. The quality of the fit was quantified using the coefficient of determination (*R*^2^), and the identified material parameters together with the corresponding *R*^2^ values are reported in Table 1.

**Table 1:** Parameters describing the orthotropic material of each one of the five zones

Zone	*C*_10_ (MPa)	${k}_{1}$(MPa)	${k}_{2}$	*R*^2^-longitudinal	${k}_{3}$(MPa)	${k}_{4}$	${K}_{0} (\mathrm{MPa})$	*R*^2^-circumferential
*A*	0.0226	0.00100	1.34	0.924	0.00234	1.71	0.224	0.986
*B*	0.0272	0.00200	3.34	0.988	0.00232	1.92	0.270	0.988
*C*	0.0277	0.00200	3.78	0.991	0.00922	0.885	0.275	0.985
*D*	0.0281	0.00819	3.88	0.981	0.00819	1.01	0.279	0.982
*E*	0.0223	0.00115	1.31	0.989	0.0115	0.978	0.221	0.987

### Segmentation and Meshing

Segmentation process of the cut-open aorta was carried out with the software 3DSlicer (Brigham and Women’s Hospital, Harvard University, NIH) using the command “threshold”; the commands “erase” and “paint” were also employed to clean the extracted vessel from noise. From the medical image it was not possible to clearly distinguish intima, media and adventitia layers. The resulting stereolithographic file was then imported in ANSA Pre Processor v22.0 (BETA CAE System, Switzerland) and meshed with tetrahedral linear elements. The opposite edges of the cut were meshed in order to have the same number and organization of the nodes so to guarantee a one-to-one node matching when closing the cut. The same segmentation strategy was performed to extract the stereolithographic file of the pressurized configuration in order to extract the lumen of the vessel at 120 mmHg.

A mesh sensitivity analysis was performed to determine an appropriate element size for the simulation. The closure of the cut was simulated using four different mesh sizes: 0.80, 0.65, 0.50, and 0.40 mm. The first principal strain at the end of the closure was evaluated in each case by averaging the values from the elements surrounding the one exhibiting the maximum strain in each zone. The results were then compared across all mesh sizes in terms of percentage difference. Notably, the comparison between the 0.65 and 0.50 mm meshes showed percentage differences of less than 5.0%, as summarized in Table 2. Given these results we decided to work with an element size of 0.50 mm. The resulting mesh is shown in Fig. 1c.

**Table 2:** Results of the sensitivity analysis

	Mesh size 0.80	Mesh size 0.65	Mesh size 0.50	Mesh size 0.40	%diff. (0.8–0.65)	%diff (0.65–0.5)	%diff (0.5–0.4)
Ip strain *A*	0.127	0.141	0.142	0.141	− 9.93	− 0.704	0.709
Ip strain *B*	0.0953	0.101	0.0994	0.101	− 5.64	1.61	− 1.58
Ip strain *C*	0.0423	0.045	0.0443	0.0433	− 6.00	1.58	2.31
Ip strain *D*	0.0449	0.0533	0.0533	0.0540	− 15.8	0.0	− 1.30
Ip strain *E*	0.0571	0.0676	0.0669	0.0670	− 15.5	1.05	− 0.149

### Simulations

Simulations were performed on 28 CPUs of an Intel Xeon64 with 250 GB of RAM using the commercial finite element solver LS-DYNA 971 Release 14.0 (ANSYS, Canonsburg, PA, USA). An implicit scheme with automatic time-step algorithm was applied to the simulations.

#### Residual Stress Estimation

The aorta was modeled as a hyperelastic, orthotropic material and assigned zone-specific material properties in the longitudinal and circumferential directions, as identified from the mechanical characterization. The vessel was subdivided into five axial regions of equal length, corresponding to the locations from which experimental samples were extracted. Region-specific material parameters were assigned by superimposing the computational mesh onto images of the cut sections, allowing qualitative identification of the elements associated with each region. The Holzapfel–Gasser–Ogden model [27] was used with the parameters reported in Table 1. The nodes at the ends of the aorta were spatially constrained to replicate the boundary conditions present during the experiment. The closure of the aorta was achieved by applying displacement boundary conditions to the nodes on the cut faces. In brief, each node on one face was displaced along a linear path toward its corresponding node on the opposite face, meeting halfway. From this closed configuration, the centreline of the vessel was extracted and used to calculate the opening angles of the initial open configuration by referencing the nodes along the centreline and the corresponding nodes on the cut faces at the lumen edges. Then, a tied contact was activated between the two cut surfaces. Following this, the displacement boundary conditions were released, allowing the aorta to deform freely and reach a new equilibrium configuration that inherently includes the residual stress field. The resulting closed configuration was exported and the corresponding nodes along the cut were merged to reconstruct a closed aorta geometry without the inclusion of residual stresses.

#### Pressurization Simulation

To assess the effect of residual stresses on the final shape and stress state of the aorta, the closed aorta, both accounting for and not accounting for the residual stresses, was pressurized up to 120 mmHg with a homogeneous load applied to the internal lumen. Principal stresses were extracted at 80 mmHg and 120 mmHg and compared between the two cases. In addition, the lumen geometry at 120 mmHg was extracted from both simulations. In particular, cross-sectional areas at specific locations within the cut region were considered for validating the methodology. In this regard, extracted cross sections from the simulations, i.e., models with and without residual stress, were compared with cross sections extracted from the segmented image of the artery pressurized at 120 mmHg.

## Results

### Open Configuration of the Aorta

**Fig. 3:**
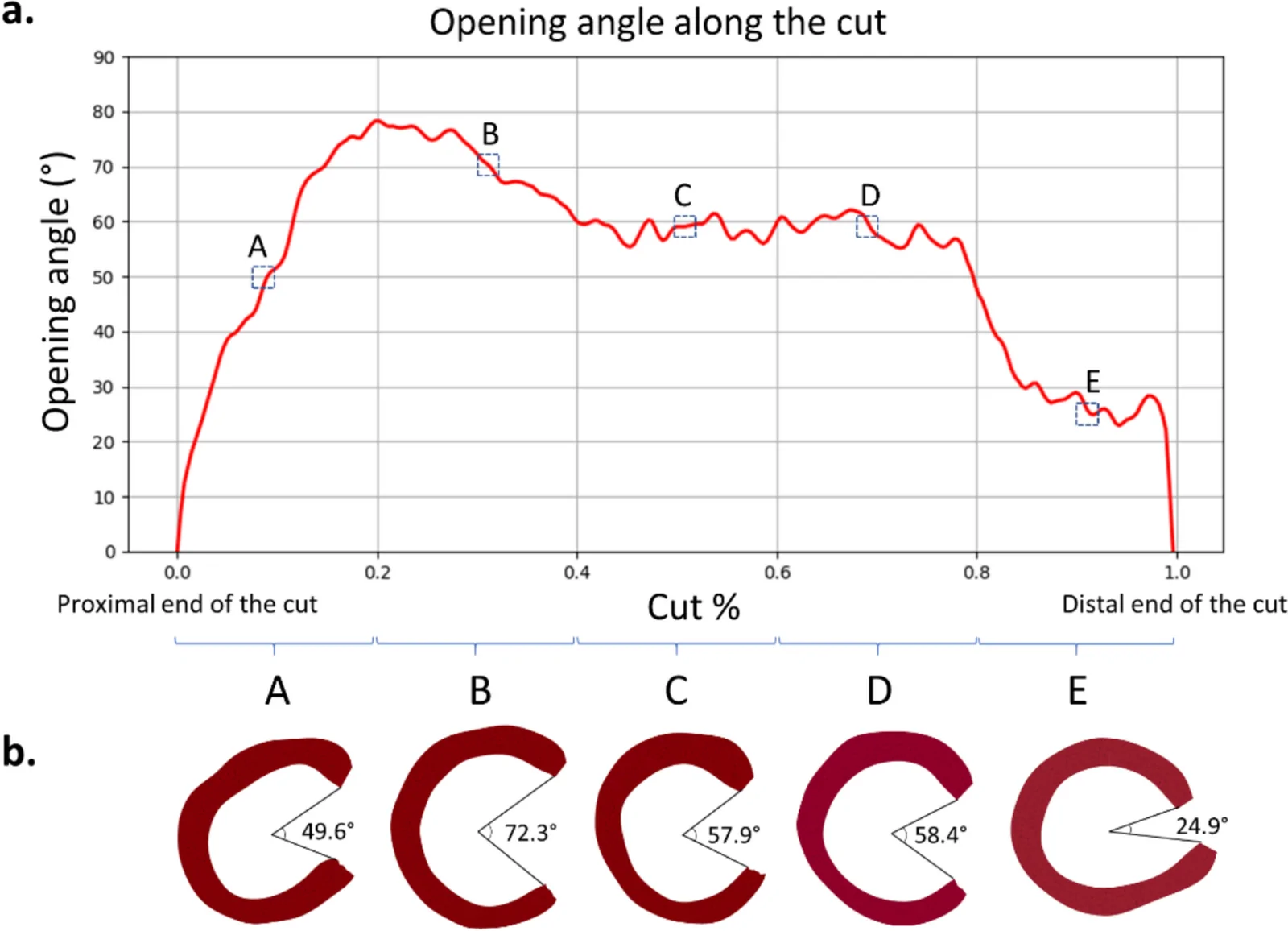
Opening angles observed after 2 h from the cut. **a** Opening angle in degrees with respect to the cut position. **b** open section in the midpoint for each one of the five zones from Fig. 2a

As soon as the aorta is longitudinally cut, it starts to open until it reaches a stable open configuration. By suspending the aorta in water and securing it at both extremities, contact with the bottom of the container was prevented, thereby avoiding any potential alteration of the resulting open configuration. Their distribution along the length of the cut is shown in Fig. 3a. Starting from the ends of the cut, the opening angle increases rapidly before stabilizing in the central region. Notably, the opening angle varies depending on location: the highest values are observed in the proximal portion of the aorta, with a peak of 78.6°, while the central region exhibits a relatively constant value of approximately 55.0° and the distal part a lower opening of about 25.0°. Sections in the midpoint of each one of the five identified zones with the corresponding opening angle are reported in Fig. 3b. In addition to the radial opening, a bending of the aorta with a restriction of the lumen was observed near the end of the cut (Fig. 1c).

### Circumferential Residual Stress Estimation

**Fig. 4:**
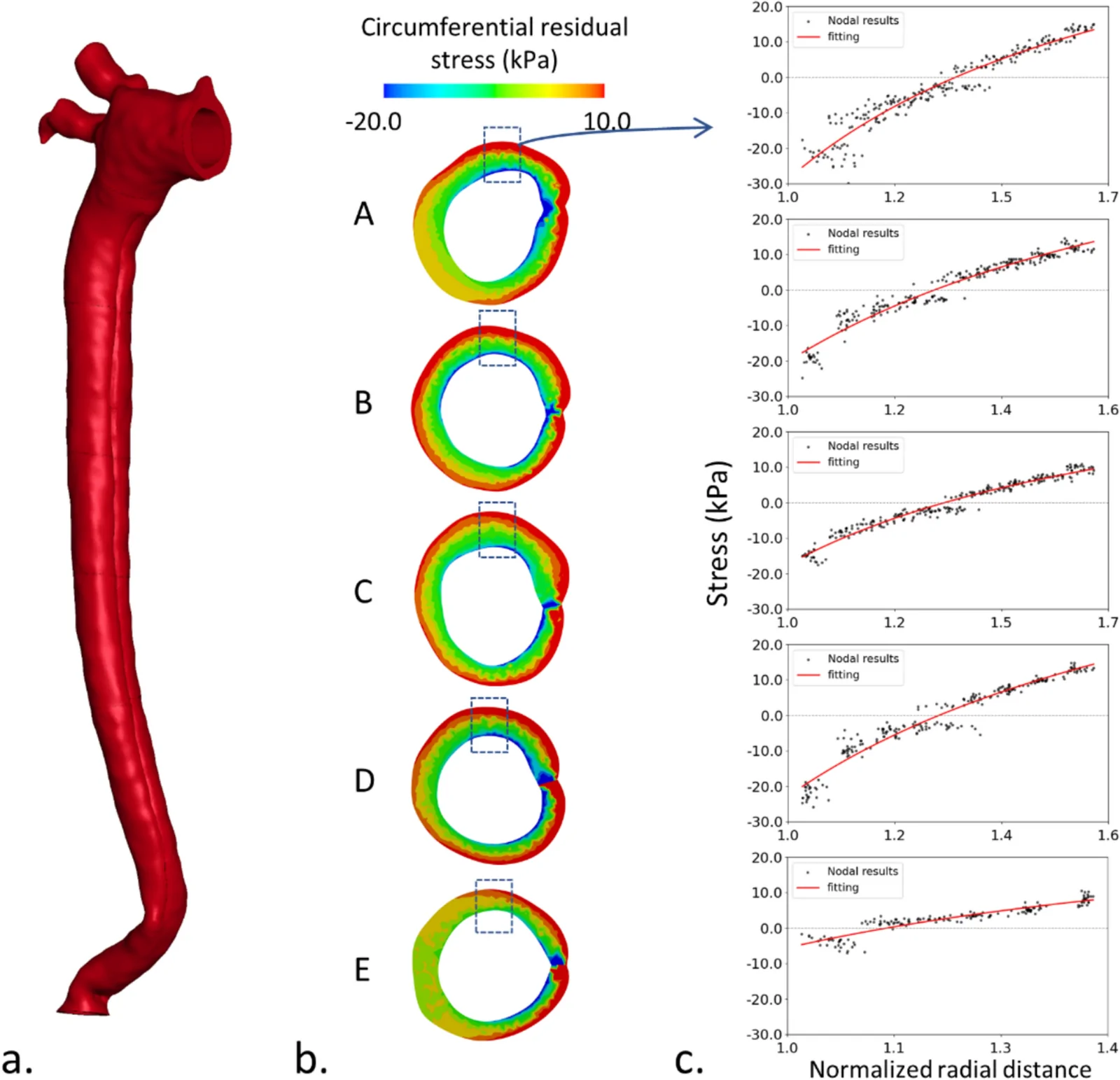
**a** Closed configuration of the vessel resulting from the simulation. **b** Contour plot of the circumferential residual stress in the midpoint section of the five identified zones and detail of sectors from which nodal results were extracted. **c** Plot of nodal circumferential residual stress extracted from nodes in blue rectangles in the anterior quadrants with respect to the normalized radial distance with respect to the lumen radius

Figure 4a shows the closed configuration of the aorta obtained from the simulation. Upon closure, the pre-existing circumferential residual stresses are re-established within the aortic wall. Their distribution in the midpoint of each of the five identified zones is shown in the contour plots in Fig. 4b, while the graphs in Fig. 4c shows how these stresses vary across the wall thickness for a representative section (rectangle in Fig. 4b) extracted from the anterior quadrant, including nodal results and a fitted curve for better visualization. Fitting was performed with the curve:

3 $$\sigma_{fitting} = \frac{a}{r} + b$$

in which *r* stands for the radial distance, while parameters *a* and *b* were determined by fitting the experimental data. Results of the fitting are reported in Table 3.

**Table 3:** Results of fitting performed for the residual stress, stress state at 80 mmHg and at 120 mmHg

		With residual stresses			Without residual stresses		
		*R*^2^	*a*	*b*	*R*^2^	*a*	*b*
Residual stress	*A*	0.9236	− 602.2	68.21	–	–	–
	*B*	0.9461	− 614.3	69.16	–	–	–
	*C*	0.9587	− 380.3	45.75	–	–	–
	*D*	0.9413	− 567.3	74.63	–	–	–
	*E*	0.8933	− 301.4	50.08	–	–	–
80 mmHg	*A*	0.9203	− 614.1	90.13	0.6967	556.1	− 25.63
	*B*	0.3229	− 121.5	37.22	0.8393	752.5	− 51.19
	*C*	0.5476	92.89	12.41	0.9567	626.6	− 46.36
	*D*	0.2991	113.8	11.63	0.8716	889.3	− 82.48
	*E*	0.7908	776.1	− 59.86	0.8187	776.1	− 139.9
120 mmHg	*A*	0.4384	− 543.4	104.5	0.7753	3112	− 204.4
	*B*	0.5731	557.1	− 4.173	0.8431	1921	− 129.4
	*C*	0.9293	658.1	− 26.70	0.9577	1396	− 99.67
	*D*	0.7985	949.2	− 57.52	0.8724	2205	− 186.6
	*E*	0.8599	3408	− 334.4	0.8366	4463	− 474.3

In all the sections, compressive stresses are observed near the lumen, while tensile stresses develop along the outer wall. In correspondence of the attached cut faces, stresses do not show this distribution, likely due to numerical noise caused by the contact algorithm. In the first and last analyzed sections near the proximal and distal ends of the cut, the residual stresses do not fully develop in the whole section due to the proximity of the non-cut zone affecting the deformation.

### Pressurization

#### Lumen Cross-Sectional Area at 120 mmHg

**Fig. 5:**
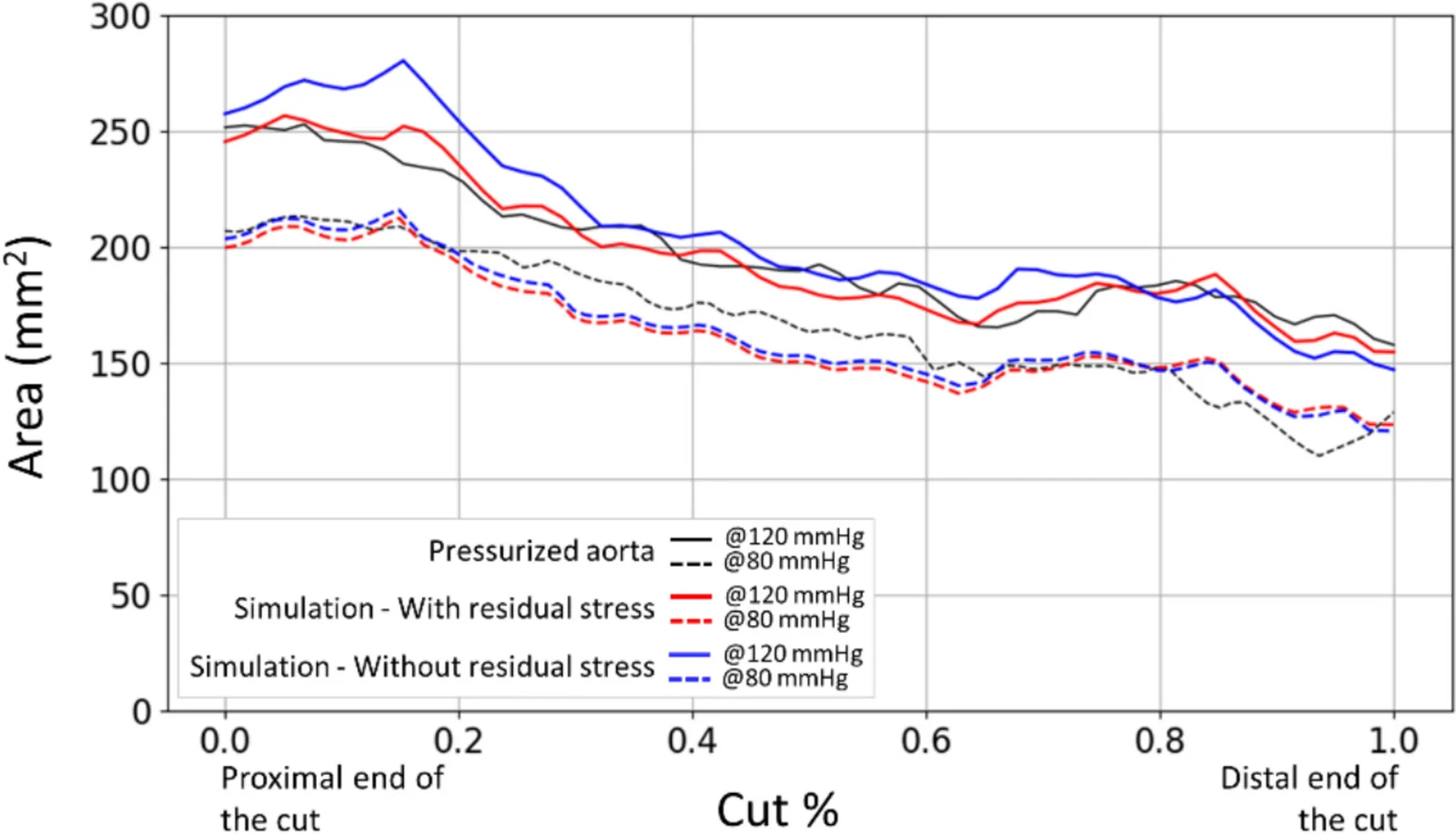
Cross-sectional lumen areas along the cut at systole

The cross-sectional areas of the vessel lumen in the cut zone at 120 mmHg with respect to the percentual position of the centreline is shown in Fig. 5. Good agreement was observed between the experimentally pressurized aorta and the numerical simulations. When residual stress was included, the average absolute percentage difference between experimental and simulated lumen cross sections was 5.99 ± 4.13 % at 80 mmHg and decreased to 2.85 ± 1.73 % at 120 mmHg. At 80 mmHg, simulations neglecting residual stress showed discrepancies of comparable magnitude (4.93 ± 3.64 %), whereas at 120 mmHg the mismatch increased to 5.57 ± 3.49 %, indicating a reduced agreement at higher pressure levels. A direct comparison between the two simulated configurations shows that the model without residual stress predicts a slightly larger lumen. The absolute percentage difference between the two cases was 1.72 ± 0.54 % at 80 mmHg and increased to 4.75 ± 1.74 % at 120 mmHg.

#### First Principal Stress Comparison

**Fig. 6:**
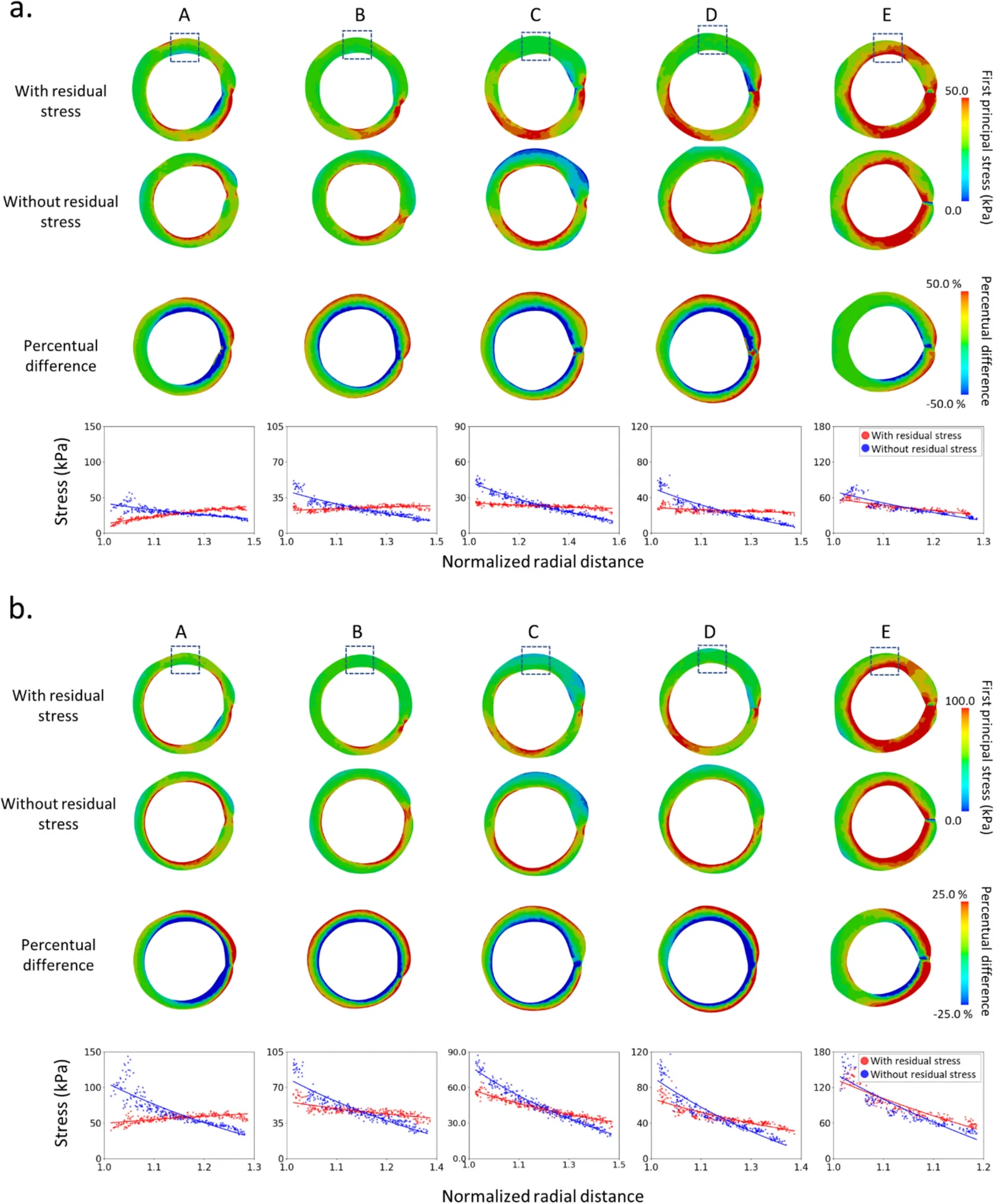
**a** Contour plot of 80 mmHg first principal stress in the midpoint of each of the five identified zones with and without residual stress. The percentual difference between the two cases were evaluated, and nodal stress was plotted as a function of the normalized radial distance relative to the lumen radius. **b** Contour plot of 120 mmHg first principal stress in the midpoint of each of the five identified zones with and without residual stress, percentual difference of the two cases and plot of nodal stress with respect to normalized radial distance with respect to lumen radius [25]

The stress state for the two simulated cases was evaluated at 80 and 120 mmHg. In both cases, the first principal stress was found to align with the circumferential direction. These results are presented in Fig. 6, which includes the contour plots of the stress distribution and the percentage difference between the two cases at 80 mmHg (Fig. 6a) and 120 mmHg (Fig. 6b), measured at the midpoint of each zone. To better illustrate the development of stress across the wall thickness and the differences between the two conditions, nodal stress values with respect to the radial distance from the centreline for a specific sector in the two simulated cases is also reported. The curve resulting from the fitting of these nodal results with the function in Eq. 2 is also shown; results of the fitting are reported in Table 3. In the case without residual stresses, peak stresses occur at the inner lumen and gradually decrease toward the outer wall according to Laplace law for pressurized tubes. In addition, the magnitude of stress is influenced by patient-specific anatomical features, in particular by vessel thickness, regions with thinner walls exhibit higher stress concentrations. Conversely, when residual stresses are included, the stress distribution becomes more uniform across the thickness, especially at lower pressure levels. In this case, the difference in stress between the inner and outer walls is reduced up to the 73.40% at 80 mmHg and the 52.62% at 120 mmHg in the analyzed regions. The percentage difference between the two cases is calculated using the following formula:

4 $$Percentual difference = 100\frac{{\sigma_{{{\mathrm{Re}} sidual stress}} - \, \sigma_{Without residual stress} }}{{\sigma_{{{\mathrm{Re}} sidual stress}} }}$$

This comparison shows that the overall distribution remains consistent across different pressure levels: residual stresses lead to lower stress values at the inner lumen and higher values at the outer wall. Notably, the peak percentage differences reach approximately ± 50% at 80 mmHg and ± 25% at 120 mmHg.

## Discussion

To the best of our knowledge, this work represents the first attempt in the literature to incorporate localized circumferential residual stresses in a finite element blood vessel model while accounting for anatomical detail and locally defined mechanical properties. First, an *in vitro* experiment was carried out on a single pig aorta within 12 h after the excision from the dead donor. CT images of pressurized pig aorta configurations at 80 and 120 mmHg were collected. The presence of circumferential residual stresses in the porcine aorta was then evaluated through observation of its opening behavior following a longitudinal cut. The medical image of the open configuration was collected 2 h after the cut and samples from different heights of the vessel were extracted to characterize circumferential and longitudinal mechanical properties. Secondly, a finite element simulation was performed to estimate the circumferential residual stresses by virtually closing the segmented open configuration; in addition, the influence of residual stresses on the pressurized state was assessed by simulating the subsequent pressurization.

The configuration recorded 2 h after the incision was considered to represent the stress-free state of the vessel. This time interval was deemed sufficient to allow the aorta to fully relax and stabilize. Previous studies have employed shorter rest times to estimate the stress-free configuration: Fung *et al.* [1] used 15 min, Peña *et al.* [28] adopted 30 min, Sokolis *et al.* [10] used 1 h, and Labrosse *et al.* [26] with 20 min. In particular, Sokolis *et al.* investigated this aspect in detail and demonstrated that approximately 95% of the residual stress is released within the first h following the cut, addressing the opening after that time span as related to viscoelastic properties of the vessel. This observation justifies the much higher release time observed in other works such as Holzapfel *et al.* [6] and Zhang *et al.* [21] with 16 h, Amabili *et al.* [29] with 6 h.

In addition to the radial opening, in our experiment a noticeable bending of the aorta was observed at the ends of the vessel, indicating the presence of complex deformation patterns beyond the vessel opening. This phenomenon could be explained with the buckling, phenomenon of mechanical instability caused by the compressive load on the internal side of the vessel that tend to close the internal lumen under no pressure similarly to what we observed (Fig. 1c). This phenomenon was already experimentally observed in previous works [7, 21] in circumferential vessel samples harvested from dead donors, and further computationally investigated [21]. In our case, we noticed the buckling of the vessel in correspondence of the end of the cut exactly where the compressive state due to circumferential residual stresses are still present in the vessel. This phenomenon has a direct influence on the results of our simulation. The open and bended configuration obtained through segmentation of the medical image represents our undeformed configuration that the vessel will try to recover given the hyperelastic description of the vessel. Hence, even with the closure of the aorta and further pressurization we were not able to recover the original closed shape of the aorta (Fig. 1c). Nevertheless, we believe the circumferential residual stresses predicted, the stress state developed in the cut zone of the vessel and the comparison performed to be reliable.

Consistent with the observations reported by Sokolis *et al.* [30], we found that opening angles vary along the vessel, depending on the location. The resulting opening angles exhibit the same trend as those reported by Sokolis *et al.* Fare clic o toccare qui per immettere il testo.[26, 30], with the opening angle decreasing progressively toward more distal regions of the intact vessel. In our approach, the measured opening angle is partially constrained by the intact regions of the vessel, particularly near the ends of the cut. As shown in Fig. 4, this is reflected in the circumferential stress distribution, where residual stresses are notably limited near the cut boundaries. However, in the central region of the cut, our simulation successfully reproduced a typical residual stress pattern [18, 24, 25], with compressive stress near the lumen and tensile stress toward the outer wall. The circumferential residual stress distribution with respect to the radial distance seems to align with the ones already described in the literature [2, 25]. We obtained residual stress values that change along the cut due to changes in material properties, vessel thickness and opening angles. While simulating closure using a tied contact algorithm between opposing cut faces introduces some numerical artifacts, especially at the cut interface, these remain localized and do not significantly affect the stress state in the rest of the vessel.

Due to the bending observed after stress release, which persisted under simulated pressurization, the analysis was limited to the lumen expansion within the cut region. Overall, good agreement was observed between experimental measurements and both numerical models. At 80 mmHg, simulations with and without residual stress predicted comparable lumen areas, whereas at 120 mmHg the inclusion of residual stress resulted in a closer agreement with the experimental configuration. The differences between the two numerical models can be explained by the stress redistribution induced by residual stress. Circumferential residual stress leads to a more uniform circumferential stress distribution across the vessel wall without altering the mean stress level (Fig. 6), resulting in similar macroscopic compliance, particularly at diastolic pressure where the transmural stress gradient is mild in both cases. At systole, the absence of residual stress produces a larger transmural stress gradient, which is associated with a reduced effective compliance and slightly larger predicted lumen areas. The remaining discrepancy with CT-derived lumen areas at diastole may also be influenced by an overestimation of arterial stiffness at low strains, likely related to the experimental characterization protocol. Although the effect of residual stress on lumen area was modest, its inclusion affects local stress distributions and may be relevant in computational studies focused on device–vessel interactions.

Comparing the stress results in the pressurization with and without the inclusion of circumferential residual stress, we obtained important differences in the first principal stress. Compressive residual stresses intervene by reducing the peak stress acting on the lumen while tensile residual stress increase the stress on the outer wall. This effect overall results in a more homogenous stress state across the thickness with a lower difference between lumen and external wall stress when residual stresses are taken into account (Fig. 6). This aspect was already observed and discussed in [23, 24, 29]. The homogenizing effect of the residual stress was found to affect differently the stress on the vessel based on the opening angle of that specific section other than the vessel thickness (Fig. 6). In all the percentual differences plot reported in Fig. 6, it is interesting to notice that all the plots show the same qualitative distribution of the circumferential residual stress (Fig. 4). These observations are confirmed by the fitting results (Table 3). The pressurization fitting reveals distinct regional behaviors. In the three central sections, the data are well described by a Laplace-based model, indicating that internal pressure dominates the effective wall loading at a first-order level. This agreement should be interpreted phenomenologically, given that the assumptions underlying the Law of Laplace are not strictly met in the aorta. When residual stress is included, the fitting quality decreases, as evidenced by lower *R*^2^ values, suggesting that Laplace-based models are insufficient to capture the combined effects of pressurization and residual stress. At 120 mmHg, pressurization becomes dominant over residual stress, improving the fit. In the distal section, stress is well fitted even at 80 mmHg, likely due to a reduced opening angle (possibly limited by bending), which minimizes residual stress influence. In contrast, in the proximal section, residual stress remains dominant even at 120 mmHg, as indicated by the negative value of parameter *a*. A fundamental aspect of this result is related to the estimation of the maximum stress. In fact, finite element simulation in in-silico medicine is often used to investigate the risk of patient-specific vessel rupture or damage in response to physiological loading and medical devices. With our results, we showed that peak stress values are reduced of up to 50.0 and 25.0% at diastolic and systolic pressures, respectively, in residually stressed vessel (Figure 6); neglecting residual stress could result in overestimating the risk of vessel damages. The proposed strategy could be translated to human vessels which exhibit a similar opening pattern, as confirmed by previous findings in the literature [8, 10]. Based on this, we expect that results comparable to those presented in this study would be obtained for healthy human vessels. Interestingly, Wang and Gleason [15] showed that incorporating longitudinal residual strains, quantified layer-specifically in porcine coronary and carotid arteries, resulted in a three-fold reduction of the transmural circumferential stress gradient at 120 mmHg compared to models neglecting these strains. In contrast, in the present imaging-based reconstruction, the homogenizing effect induced by circumferential residual stresses at the same pressure level was less pronounced, as the mechanical response became increasingly dominated by internal pressurization. It is therefore conceivable that inclusion of axial residual strains could further enhance stress homogenization and peak stress attenuation, potentially preserving these effects even at systolic pressure levels. It should be emphasized, however, that the findings of Wang and Gleason [15] are not for the aorta for which the distribution and magnitude of residual strain may vary. In addition, their results were obtained within an idealized cylindrical, homogeneous, and analytical framework based on animal data, and their direct translation to patient-specific geometries remains to be established. An interesting direction for future work would be to investigate how pathologic alterations of the vessel wall may affect the residual stress state. This work is not exempt from limitation. First of all, we are aware that this strategy for the inclusion of residual stresses does not allow for the complete opening of the vessel, necessarily dampening the resulting residual stresses, the estimation of axial pre-stretches, layer-specific, or quadrant-specific residual stresses. At the same time, we are aware that this is the only available option to directly estimate residual stresses on pig anatomy while preserving the integrity of the anatomical structures. Regarding axial residual stresses, their inclusion was not compatible with the imaging-based workflow adopted in this study, which aimed to preserve patient-specific anatomical features following the longitudinal cut to unveil the distribution of residual circumferential strains. Moreover, even though the adoption of a single aorta is a strong limitation, the proposed study should be considered as a pilot study and a proof of concept about the novel methodology herein presented, several further studies will be proposed to ensure the quality and the reliability of the method other than finding a method to enrich the methodology with patient-specific longitudinal residual strain to further investigate circumferential and longitudinal stress interplay as hypothesized by Sokolis *et al* [11] and analytically demonstrated by Wang and Gleason [15]. As a second consideration, we worked with a homogenous model of the vessel without considering layer-specific residual stresses. This choice comes from the impossibility to distinguish between intima, media, and adventitia layers during the segmentation process of the extracted image other than of course characterizing opening angles of the three layers without damaging our samples which would make our residual stress estimation strategy unfeasible. Also, differently from other works in the literature, our aorta was immersed in plain water instead of saline solution. Lastly, with this work we intended to spread light on the effect of neglecting residual stresses rather than presenting a method to *in vivo* identify the parameters for their inclusion; we are aware that this method could not be applied when modeling *in vivo* vessels extracted through medical images.

In this study, we aimed to investigate the impact of including circumferential residual stress in finite element modeling of blood vessels. We adopted a strategy different from those commonly found in the literature, with the goal of more accurately accounting for localized features, such as varying wall thickness and the integration of spatially defined mechanical properties. Our observations revealed that the vessel opening following the longitudinal cut is not uniformly distributed, and this asymmetry has a direct influence on the resulting residual stress field. A comparison between simulations with and without residual stress showed differences in the mechanical response of the vessel. First, we observed higher expansion of the vessel when pressurized without the inclusion of circumferential residual stresses, aspect that could affect the response of implantable device. Second, the inclusion of residual stresses led to a more homogeneous stress distribution, particularly by reducing the stress peaks near the inner lumen. These findings suggest that excluding residual stress in finite element models may result in inaccurate estimations of the mechanical risks to which the patient is exposed, potentially compromising the predictive power of such simulations.
